# Antagonistic cotranscriptional regulation through ARGONAUTE1 and the THO/TREX complex orchestrates *FLC* transcriptional output

**DOI:** 10.1073/pnas.2113757118

**Published:** 2021-11-17

**Authors:** Congyao Xu, Xiaofeng Fang, Tiancong Lu, Caroline Dean

**Affiliations:** ^a^Department of Cell and Developmental Biology, John Innes Centre, Norwich NR4 7UH, United Kingdom

**Keywords:** RNA processing, ARGONAUTE1, THO/TREX complex, cotranscription

## Abstract

Quantitative transcriptional control is essential for physiological and developmental processes in many organisms. Transcriptional output is influenced by cotranscriptional processes interconnected to chromatin regulation, but how the functions of different cotranscriptional regulators are integrated is poorly understood. The *Arabidopsis* floral repressor locus *FLOWERING LOCUS C* (*FLC*) is cotranscriptionally repressed by alternative processing of the antisense transcript *COOLAIR.* Proximal 3′-end processing of *COOLAIR* resolves a cotranscriptionally formed R-loop, and this process physically links to a histone-modifying complex FLD/SDG26/LD. This induces a chromatin environment locally that determines low transcription initiation and a slow elongation rate to both sense and antisense strands. Here, we show that ARGONAUTE1 (AGO1) genetically functions in this cotranscriptional repression mechanism. AGO1 associates with *COOLAIR* and influences *COOLAIR* splicing dynamics to promote proximal *COOLAIR*, R-loop resolution, and chromatin silencing. Proteomic analyses revealed physical associations between AGO1, subunits of RNA Polymerase II (Pol II), the splicing-related proteins—the spliceosome NineTeen Complex (NTC) and related proteins (NTR)—and the THO/TREX complex. We connect these activities by demonstrating that the THO/TREX complex activates *FLC* expression acting antagonistically to AGO1 in *COOLAIR* processing. Together these data reveal that antagonistic cotranscriptional regulation through AGO1 or THO/TREX influences *COOLAIR* processing to deliver a local chromatin environment that determines *FLC* transcriptional output. The involvement of these conserved cotranscriptional regulators suggests similar mechanisms may underpin quantitative transcriptional regulation generally.

## Significance

RNA processing generally occurs as transcripts are being produced and the concomitant cotranscriptional processes are interconnected with chromatin regulation. These cotranscriptional mechanisms quantitatively influence transcriptional output. At the *Arabidopsis* gene *FLC*, repression involves alternative processing of *FLC* antisense transcripts linked to delivery of a local chromatin environment that determines *FLC* transcription initiation and elongation rate. Here, we show that AGO1, a factor known predominantly for its role in posttranscriptional gene silencing, is involved in this cotranscriptional repression mechanism. Conserved cotranscriptional regulators, the THO/TREX complex and NTC components of the activated spliceosome, physically associate with AGO1. Our analysis suggests that alternative interactions of cotranscriptional regulators with the RNA Pol II–spliceosome link RNA processing and chromatin modification to quantitatively regulate transcriptional output.

RNA-mediated chromatin regulation has emerged as a key mechanism in gene regulation in eukaryotes (1). It is well-established how small RNAs (sRNAs) regulate chromatin silencing in heterochromatin formation in fission yeast, plants, and animal germlines (2). Long noncoding RNA (lncRNA) also plays pivotal roles in chromatin regulation. A classic example is the X inactive specific transcript (*Xist*)–mediated X chromosome inactivation in female mammals (3). One area, however, where our understanding is poor is how cotranscriptional processing of nascent transcripts can dynamically modulate transcriptional output through the regulation of chromatin.

Plants as sessile organisms have evolved to rapidly respond to environmental changes and thus provide an ideal system to dissect such mechanisms. In *Arabidopsis thaliana*, expression of the floral repressor locus *FLOWERING LOCUS C* (*FLC*) plays a central role in determining reproductive strategy—overwintering or rapid cycling—with considerable consequence on reproductive success in different climates (4–6). Regulators of *FLC* expression have been identified through genetic screens for early- and late-flowering plants. Remarkably, the majority of factors identified are general cotranscriptional regulatory factors involving RNA processing and chromatin regulation. For example, activators of *FLC* expression include homologs of yeast RNA polymerase II–associated factor 1 complex (Paf1-complex), SET-domain proteins, SWR1 chromatin-remodeling complex, and H2B monoubiquitination mediators (reviewed in ref. 7). Functioning antagonistically to these are repressors of *FLC* expression: conserved RNA binding proteins (FCA, FPA, and FLK), RNA 3′-end processing and splicing factors, and chromatin modifiers. These repressors were grouped into the autonomous pathway that regulates *FLC* through an RNA-mediated chromatin-silencing mechanism (reviewed in ref. 8). Our current understanding of this mechanism is that the RNA-binding protein FCA concentrates 3′-end processing factors in nuclear condensates and promotes proximal polyadenylation of a set of antisense long noncoding transcripts transcribed from *FLC*, called *COOLAIR* (9, 10). These 3′-end processing factors dynamically interact with a histone-modifying complex FLD/SDG26/LD, thereby removing H3K4me1 over the *FLC* gene body. The act of promoting proximal polyadenylation of *COOLAIR* resolves an R-loop, and this is essential to trigger the histone demethylation-induced chromatin silencing (11). This process creates a chromatin environment that reduces transcriptional initiation and slows the elongation rate, contributing to the quantitative repression of *FLC* expression (12, 13). In summary, the quantitative output of transcription at *FLC* is set by antagonistic functions of activators and repressors in a mechanism involving RNA/lncRNA processing and chromatin regulation. However, how these antagonistic functions converge and how quantitative output is modulated had not been previously addressed.

Argonaute proteins are well-known effectors in widely conserved RNA interference (RNAi) pathways. AGO proteins loaded with sRNAs form the core of RNA-induced silencing complexes that function in posttranscriptional gene silencing, either through cleavage of target RNA or translational inhibition, both of which mainly occur in the cytoplasm (14). AGO proteins also engage in transcriptional gene silencing in the nucleus, triggering epigenetic modifications on chromatin (15). In recent years, emerging evidence has demonstrated AGOs function more widely in gene regulation including transcriptional activation, RNA splicing, DNA repair, and chromatin topology (16–23). There are 10 AGOs in *Arabidopsis*, with diverse functions and association with different sRNA species. *Arabidopsis* AGO1 is conventionally considered to work predominantly with microRNAs (miRNAs) in RNAi (24), but recent evidence has shown AGO1 undergoes nucleocytosolic shuttling and associates with chromatin in response to ultraviolet light, plant hormones, and stress (25–28). These data suggest *Arabidopsis* AGO1 may play a much wider role in gene regulation.

Here, we report AGO1 as a player in the *COOLAIR*-mediated *FLC* repression mechanism. We find that AGO1 physically associates with the FLD/SDG26/LD complex. Genetic analysis confirmed the AGO1 involvement in FCA-mediated *FLC* repression, and we show AGO1 associates with antisense RNA *COOLAIR* and influences *COOLAIR* splicing. Proteomics analyses revealed a close relationship between AGO1, the THO/TREX and NTC/NTR complexes, and RNA Polymerase II (Pol II). We show the THO/TREX complex functions antagonistically with AGO1 on *COOLAIR* processing and thus *FLC* regulation. The detailed dissection of the *FLC/COOLAIR* transcriptional unit has enabled an understanding that differential assembly of cotranscriptional machineries with RNA Pol II results in changes in RNA processing that feeds back to alter local chromatin environment and thus influence subsequent transcriptional output.

## Results

### AGO1 Functions in the Same Genetic Pathway as FCA and FLD to Repress *FLC*.

**Fig. 1.**
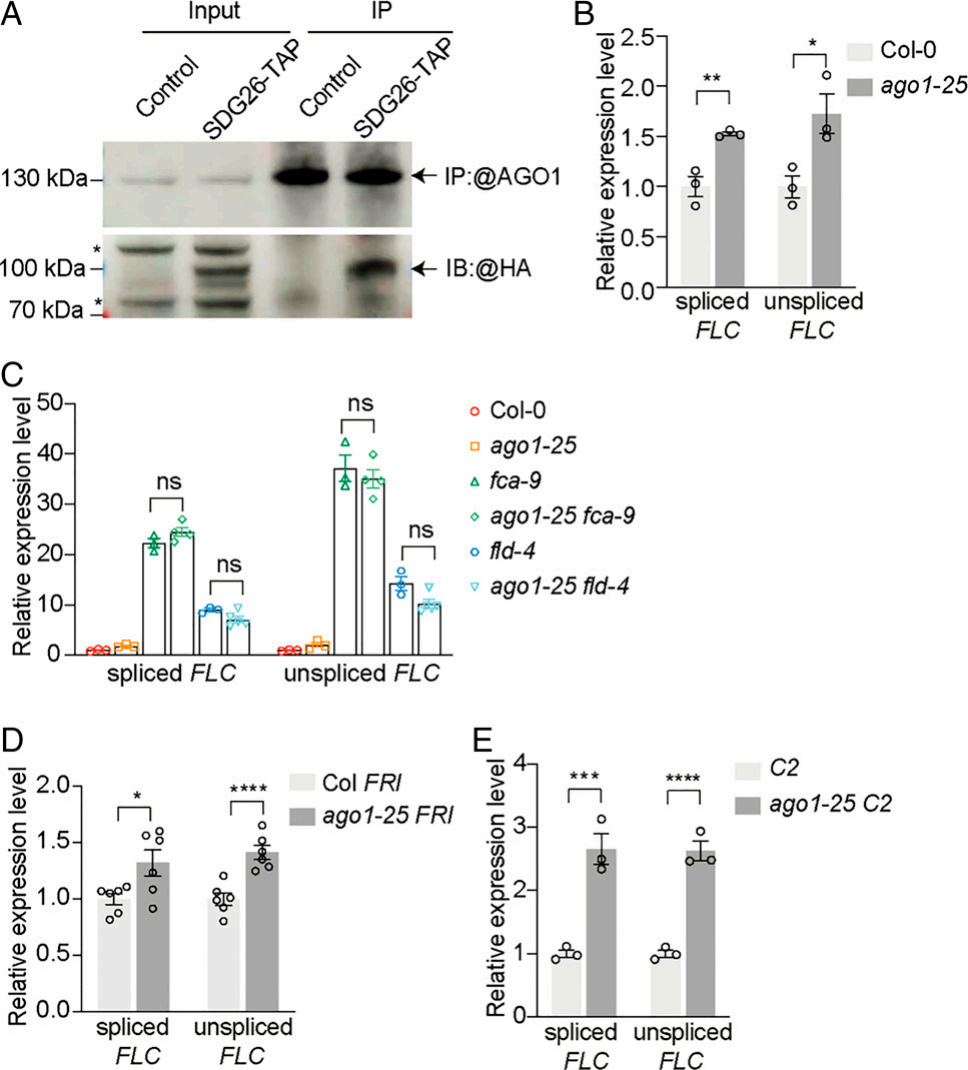
AGO1 genetically functions with FCA and FLD to repress *FLC.* (*A*) Co-IP in nuclear extracts from wild-type seedlings (Control) or seedlings expressing SDG26-TAP. IP: immunoprecipitation; IB: immunoblot. Asterisk indicates nonspecific signal. (*B*) Expression of spliced and unspliced *FLC* relative to *UBC* in Col-0 and *ago1-25* seedlings. Data are normalized to wild-type Col-0. Data are presented as the mean ± SEM (*n* = 3). Asterisks indicate significant differences between the indicated plants (**P ≤ 0.007136, *P ≤ 0.031062, two-tailed *t* test). (*C*) Expression of spliced and unspliced *FLC* relative to *UBC* in various genotypes. Data are normalized to Col-0. Data are presented as the mean ± SEM (*n* = 3 to 5). Two-tailed *P* value from multiple *t* test corrected by Holm–Sidak method; ns, not significant. (*D*) Expression of spliced and unspliced *FLC* relative to *UBC* in Col *FRI* and *ago1-25 FRI* seedlings. Data are normalized to Col *FRI.* Data are presented as the mean ± SEM (*n* = 6). Asterisks indicate significant differences between the indicated plants (*P ≤ 0.029432, ****P ≤ 0.000551, two-tailed *t* test). (*E*) Expression of spliced and unspliced *FLC* relative to *UBC* in C2 and *ago1-25 C2* seedlings. Data are normalized to *C2.* Data are presented as the mean ± SEM (*n* = 3). Asterisks indicate significant differences between the indicated plants (***P ≤ 0.002662, ****P ≤ 0.000588, two-tailed *t* test).

Interactors of the FLD/SDG26/LD complex have been identified using mass spectrometric analyses (12). A high peptide count for AGO1 was found in the SDG26-GFP affinity purification (*SI Appendix*, Fig. S1). Additionally, unique peptides of AGO1 were detected in cross-linked nuclear immunoprecipitation of SDG26-GFP (*SI Appendix*, Fig. S1). The interaction between AGO1 and SDG26 was further confirmed by coimmunoprecipitation (co-IP) using nuclear extracts from a stable transgenic line expressing SDG26 fused to a FLAG-HA TAP tag (SDG26-TAP) (Fig. 1*A*). The physical interaction between AGO1 and the *FLC* repressor complex FLD/SDG26/LD prompted us to investigate the role of AGO1 in *FLC* regulation. The FLD/SDG26/LD complex works downstream of FCA to repress *FLC* in the autonomous pathway. We therefore asked whether AGO1 functions in the same genetic pathway.

Since a complete loss of AGO1 function is sterile we exploited a weak allele *ago1-25;* this is slightly late-flowering (29) and confers increased *FLC* expression (Fig. 1*B*), similar to loss-of-function mutations of the autonomous pathway. *ago1 fca* and *ago1 fld* double mutants did not show any additive effect on *FLC* expression (Fig. 1*C*); however, an additive effect was observed when *ago1* was combined with *FRIGIDA (FRI*), a strong activator of *FLC* (Fig. 1*D*). This indicates AGO1 functions in the same genetic pathway as FCA and FLD, but independently of FRI. To further investigate the role of AGO1 in *FLC* repression, we introduced *ago1-25* into a genotype (named *C2*) carrying FRI and overexpressing FCAγ that we have used to successfully screen components required for FCA in *FLC* regulation (9, 10, 30, 31). *FLC* was significantly derepressed in *C2 ago1-25* compared to *C2* (Fig. 1*E*). Neither the native FCA protein level nor the overexpressed FCAγ was affected by the *ago-25* mutation (*SI Appendix*, Fig. S2). These data collectively demonstrate AGO1 is required for FCA-mediated *FLC* repression. AGO1 harbors an Asp-Glu-Asp-His/Asp (DEDH/D) catalytic tetrad, which is commonly used for slicing target messenger RNA (mRNA) in *Arabidopsis* (24, 32). We therefore asked whether the slicer activity of AGO1 is required for repression of *FLC*. We analyzed *FLC* expression in a set of transgenic lines either expressing wild-type slicer or deficient slicer in *ago1-25* (33). The derepression of *FLC* in *ago1-25* is only complemented by introducing a wild-type slicer (*SI Appendix*, Fig. S3), suggesting that slicer activity or an intact slicer domain is required for AGO1-mediated *FLC* repression.

### AGO1 Binds COOLAIR Transcripts and Influences COOLAIR Splicing.

**Fig. 2.**
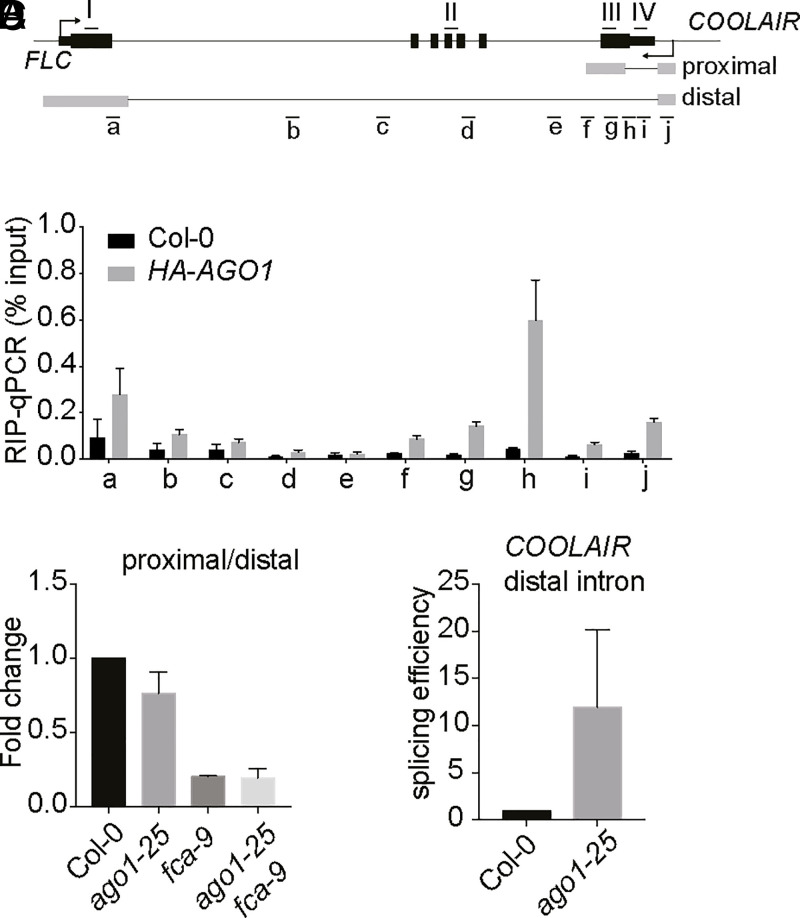
AGO1 associates with *COOLAIR* and influences *COOLAIR* processing. (*A*) Schematic diagram showing *FLC* gene structure and *COOLAIR* transcripts. Black and gray boxes represent *FLC* and *COOLAIR* exons, respectively. Black and gray lines represent *FLC* and *COOLAIR* introns, respectively. The arrow indicates the transcription start site (TSS). Short black lines with letters underneath indicate positions of amplicons in qPCR amplification. (*B*) RIP–qPCR analyzing HA-AGO1 enrichment on nascent *COOLAIR* transcript. Wild-type Col-0 was used as background control. The *x* axis corresponds to the fragments shown in *A*. Data are mean ± SD (*n* = 3). (*C*) The ratio of proximal-to-distal isoforms of *COOLAIR* transcripts (refer to the schematic in *A*) in various genotypes relative to Col-0. Data are mean ± SEM (*n* = 3). (*D*) The splicing efficiency of distal intron (spliced/unspliced) determined through chromatin-bound RNA analysis. Data are normalized to Col-0. Data are mean ± SD (*n* = 3).

AGO1 was reported to directly bind to chromatin in the *Arabidopsis* genome to positively regulate gene expression, especially in response to hormone and stress stimuli (28). However, this does not seem to be the mechanism by which AGO1 regulates *FLC* for two reasons: First, *FLC* is repressed rather than activated by AGO1; second, chromatin immunoprecipitation (ChIP) sequencing data in this report did not suggest *FLC* chromatin is enriched for AGO1. Instead, the physical association and the genetic requirement of AGO1 in the autonomous pathway of *FLC* regulation prompts us to investigate a direct role of AGO1 in this antisense RNA-mediated chromatin silencing pathway. We performed RNA immunoprecipitation (RIP) in a transgenic line expressing hemagglutinin (HA)–tagged AGO1. Intriguingly, AGO1 associates with specific segments of *COOLAIR*, especially at a region spanning the exon–intron junction in the proximally polyadenylated *COOLAIR* (Fig. 2 *A* and *B*). This specific occupancy of AGO1 on *COOLAIR* prompted us to test whether AGO1 influences *COOLAIR* processing. We detected a reduced proximal-to-distal ratio of *COOLAIR* in *ago1-25*, which is epistatic to *fca-9* (Fig. 2*C*). This suggested AGO1 works with FCA to promote proximal *COOLAIR*. Argonaute proteins have been documented to influence alternative splicing in other organisms (17–19, 34). We therefore hypothesized the shift to distal *COOLAIR* in *ago1* mutant might be due to a shift in use of the distal splice acceptor site. By analyzing chromatin-bound RNA, we indeed detected a higher ratio of spliced to unspliced distal *COOLAIR* in *ago1-25* (Fig. 2*D*). Taken together, these results suggest AGO1 targets *COOLAIR* transcript and influences splicing of *COOLAIR*.

FCA forms liquid-like nuclear condensates in vivo that concentrate 3′-end processing factors to promote efficient polyadenylation (9). Thus, we asked whether AGO1 might influence the dynamics of FCA condensates. We compared FCA-mTurquoise2 condensates in wild type and *ago1-25* mutant and found a small reduction of FCA condensate size in *ago1-25* (*SI Appendix*, Fig. S4), the subtle difference likely due to *ago1-25*’s being a weak allele. Nevertheless, the slight AGO1 stabilization of the FCA condensates may increase dwell time over the locus sufficiently to promote splicing of the proximal intron linked to 3′-end processing to promote proximal *COOLAIR* formation.

### AGO1 Promotes R-Loop Resolution and *FLC* Chromatin Silencing.

**Fig. 3.**
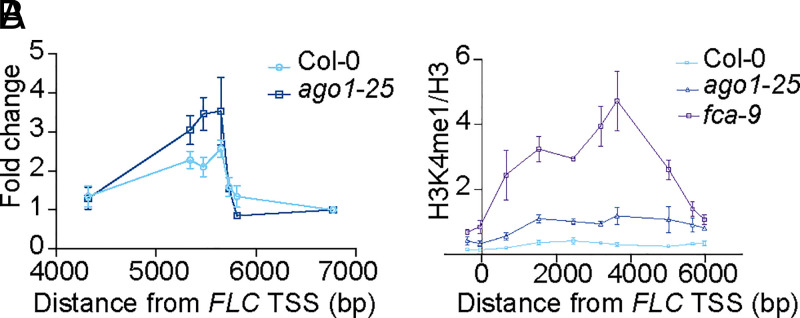
AGO1 promotes *COOLAIR* R-loop removal and *FLC* chromatic silencing. (*A*) DRIP–qPCR determining R-loop level in Col-0 and *ago1-25*. The number on the *x* axis is the distance to *FLC* TSS. Data are percentage of input normalized to a region without *COOLAIR* transcript (between 6,000 and 7,000 base pairs to *FLC* TSS). Data are presented as mean ± SEM (*n* = 3). (*B*) ChIP analysis of H3K4me1 level at *FLC* in various genotypes. The number on the *x* axis is the distance to *FLC* TSS. Data are mean ± SD (*n* = 3).

We had previously shown that processing of proximal *COOLAIR* promotes the resolution of a *COOLAIR* induced R-loop, which links to histone demethylation-induced chromatin silencing of *FLC* (11). Given AGO1 promoted proximal *COOLAIR* and repressed *FLC*, we speculated that AGO1 might also affect the R-loop dynamics. DNA/RNA immunoprecipitation (DRIP)-qPCR analysis showed the R-loop over the 3′ end of *FLC* indeed increases in *ago1-25* (Fig. 3*A*). Consistent with this, H3K4me1 over *FLC* gene body was found to increase in *ago1-25* (Fig. 3*B*). Taken together, AGO1 functions with FCA and FLD to promote *FLC* chromatin silencing, in a mechanism involving *COOLAIR* processing and R-loop resolution.

AGO1 canonically functions with miRNA and small interfering RNA (siRNA), which are mainly produced through DICER-LIKE (DCL) proteins in *Arabidopsis*. Previously, based on a T-DNA insertion mutant of *DCL3* that largely released *FLC* silencing in *35S::FCA* γ (overexpression of *FCA* γ) background we reported that DCL3 is also involved in FCA/FLD-mediated *FLC* silencing (35). This particular *dcl3* T-DNA allele suffers from posttranscriptional gene silencing due to a complex T-DNA insertion, which influences expression of other transgenes in that line (36). Introduction of an ethyl methanesulfonate–induced *dcl3* allele into the *35S::FCA* γ genotype did not result in released silencing of *FLC,* in contrast to the *ago1-25* mutant (*SI Appendix*, Fig. S5). Therefore, we conclude AGO1 is involved in FCA-mediated *FLC* silencing independently of DCL3.

### Physical Association of AGO1, RNA Pol II, and THO/TREX and NTC/NTR Complexes.

To understand the function of AGO1 in RNA processing, we investigated the proteins associated with AGO1 in the nucleus. A previous mass spectrometry analysis of nuclear AGO1 coimmunoprecipitated proteins identified SWI/SNF complex as an interactor that promotes transcription in response to stimuli (28). Other factors involved in transcription and RNA processing were also identified, including RNA Polymerase subunits, components of the NTC complex which couples RNA Pol II to splicing and the whole THO/TREX complex which links transcription with RNA processing and export (*SI Appendix*, Fig. S6*A*). We immunopurified HPR1/THO1, a component of the THO/TREX complex, from a transgenic line expressing HPR1 tagged with green fluorescent protein (GFP) (37). We were able to identify all the components of the THO/TREX complex, suggesting a successful IP. AGO1 was also identified as a coimmunoprecipitant of HPR1. Similar to the coimmunoprecipitated proteins of nuclear AGO1, RNA Pol II subunits and a plethora of factors in the NTC/NTR complex were identified, indicating a close relationship between these factors (*SI Appendix*, Fig. S6*B* and Dataset S1). We took advantage of the *FLC/COOLAIR* transcriptional unit to further understand the functional relevance of the physical associations between AGO1 and these conserved cotranscriptional regulators.

### The THO/TREX Complex Is Required for *FLC* Activation and Antagonizes AGO1 and FCA.

**Fig. 4.**
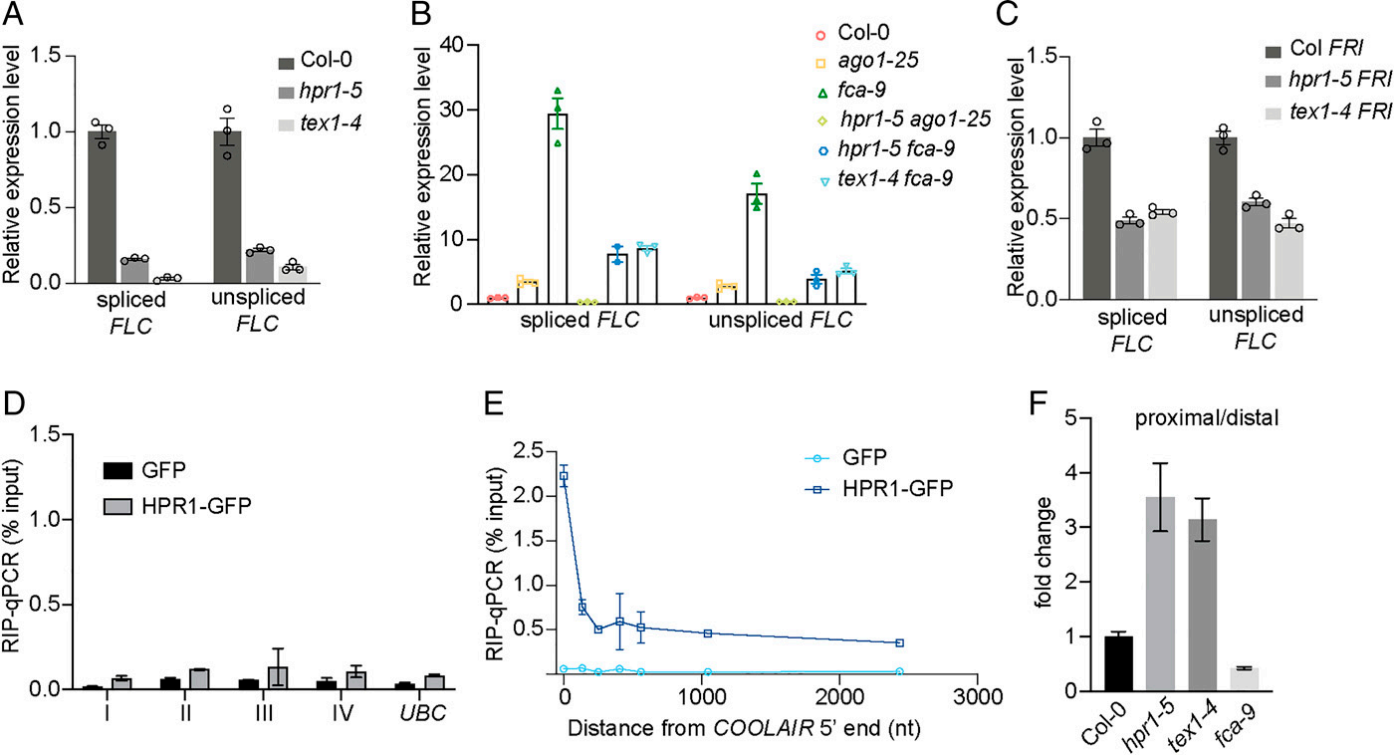
The THO/TREX complex up-regulates *FLC* and promotes distal *COOLAIR.* (*A*) Expression of spliced and unspliced *FLC* relative to *UBC* in various genotypes. Data are normalized to Col-0. Data are presented as the mean ± SEM (*n* = 3). (*B*) Expression of spliced and unspliced *FLC* relative to *UBC* in various genotypes. Data are normalized to Col-0. Data are presented as the mean ± SEM (*n* = 3). (*C*) Expression of spliced and unspliced *FLC* relative to *UBC* in various genotypes. Data are normalized to Col *FRI*. Data are presented as the mean ± SEM (*n* = 3). (*D*) RIP-qPCR analyzing HRP1-GFP enrichment on *FLC* mRNA in transgenic line expressing *HPR1-GFP.* Transgenic line expressing *GFP* alone was used as negative control. The *x* axis refers to the fragments shown in Fig. 2*A*. Data are mean ± SD (*n* = 3). (*E*) RIP-qPCR analyzing HRP1-GFP enrichment on nascent *COOLAIR.* Transgenic line expressing *GFP* was used as negative control. The *x* axis is the distance to *COOLAIR* 5′. Data are mean ± SD (*n* = 3). (*F*) The ratio of proximal-to-distal isoforms of *COOLAIR* transcripts (refer to the schematic in Fig. 2*A*) in various genotypes relative to Col-0. Data are mean ± SEM (*n* = 3).

The conserved THO/TREX complex that links multiple steps of RNA processing with RNA export has been shown to promote messenger ribonucleoprotein assembly and prevent R-loop formation in yeast and mammals (38). Given the role of AGO1 in resolving *COOLAIR*-induced R-loop, we first tested the possibility that the THO/TREX complex may prevent *COOLAIR*-induced R-loop formation. Using DRIP-qPCR analysis, we did not observe an obvious R-loop level change in the THO/TREX complex mutants *hpr1-5* and *tex1-4* (*SI Appendix*, Fig. S7). We did, however, find a reduction of *FLC* expression in these mutants (Fig. 4*A*), suggesting the THO/TREX complex activates *FLC*. Combination of these mutations with *ago1-25* or *fca-9* gave double mutants with intermediate levels of *FLC* expression (Fig. 4*B*), indicating the THO/TREX complex is required for the derepression of *FLC* in *ago1-25* or *fca-9.* We next asked whether this requirement is general for *FLC* activation, and therefore we introduced the THO/TREX mutants into the *FRI* genotype, where *FLC* is strongly activated. We found *FLC* activation is inhibited in the mutants (Fig. 4*C*), suggesting that the THO/TREX complex is also required for FRI-mediated *FLC* activation.

### The THO/TREX Complex Binds to Nascent *COOLAIR* and Promotes Production of Distal *COOLAIR.*

The general requirement of the THO/TREX complex to activate *FLC* through different pathways supports a direct cotranscriptional effect of this complex at the *FLC* locus. Therefore, we performed RIP-qPCR analysis to check the occupancy of this complex on the transcripts using a transgenic line expressing HPR1 tagged with GFP. There was relatively little enrichment of the HPR1-GFP protein with *FLC* mRNA (Fig. 4*D*), suggesting that *FLC* mRNA export is less likely to be affected. In contrast, HPR1-GFP was highly enriched with the *COOLAIR* nascent transcript (Fig. 4*E*). This suggests the THO/TREX complex mainly functions through cotranscriptional regulation of *COOLAIR*, which in turn influences *FLC* transcriptional activity in the *FLC/COOLAIR* circuit. We therefore analyzed *COOLAIR* processing and found low levels of distal *COOLAIR* in *hpr1-5* and *tex1-4* (Fig. 4*F*). The proximal *COOLAIR* was not affected in these mutants, consistent with no change in R-loop levels. In yeast, the THO/TREX complex was shown to promote transcription elongation and we speculate that this complex plays a similar mechanism at *FLC* promoting *COOLAIR* transcription elongation, and thus enhancing distal *COOLAIR* production.

## Discussion

The interconnection between RNA Pol II, cotranscriptional RNA processing, and local chromatin is poorly understood, in part due to the complex feedbacks between the different steps. We have been analyzing these interconnections through analysis of *Arabidopsis FLC* regulation. Genetic screens uncovered *FLC* activators and repressors that identified cotranscriptional regulators involved in RNA processing and chromatin modification. The *FLC* repression mechanism links RNA 3′-end processing with chromatin regulators, and the SET-domain protein SDG26 was shown to play an important role in bridging these two processes. In the work reported here, we show that AGO1 is a factor in this repression mechanism through physical interaction with FLD/SDG26/LD complex and the genetic relationship with FCA and FLD. We also find THO/TREX acts antagonistically with AGO1, thus identifying one of the convergence points of *FLC* activators and repressors. AGO1 and THO/TREX function antagonistically on *COOLAIR* processing and *FLC* regulation and both interact with the NTC components. Thus, our analysis of a developmentally important regulator has shed light on antagonistic cotranscriptional regulation that potentially underlies quantitative regulation of transcription generally.

**Fig. 5.**
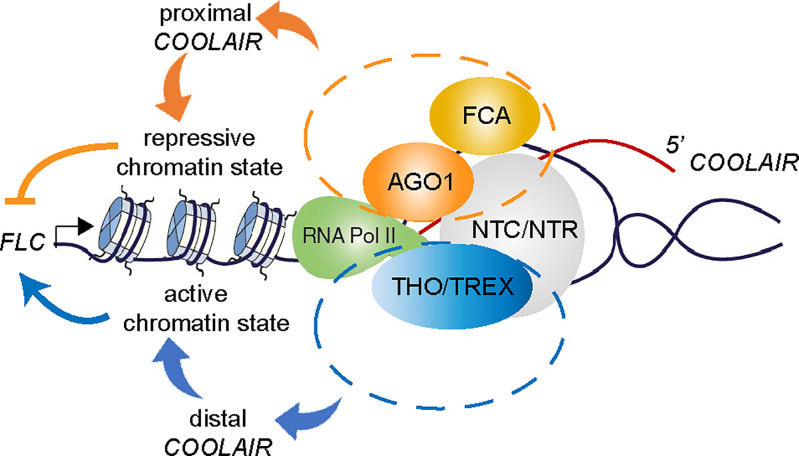
Model for how modulation of antisense RNA processing determines sense transcriptional output. Antagonistic assembly of cotranscriptional machinery with RNA Pol II influences transcript processing. During *COOLAIR* transcription, FCA, AGO1, and components of NTC/NTR link to RNA Pol II and promote proximal *COOLAIR*. THO/TREX dynamically competes for RNA Pol II to promote distal *COOLAIR*. The differential composition of the cotranscriptional regulators with the Pol II complex influences the chromatin state locally, which in turn influences the transcriptional output of the whole locus.

The multicomponent NTC/NTR complex forms part of the catalytic spliceosome and associates closely with both AGO1 and the THO/TREX complex. The physical association between AGO1 and the NTC/NTR was also revealed in other organisms. A conserved NTC/NTR component MAC7/Aquarius/EMB-4 was reported to interact with nuclear Argonaute HRDE-1 to mediate RNAi-dependent cotranscriptional silencing in a *Caenorhabditis elegans* germline (39, 40). We also find MAC7 may function together with AGO1 in *FLC* repression as *FLC* expression is derepressed in a *mac7-1* mutant (*SI Appendix*, Fig. S8). Our data show AGO1 and the THO/TREX complex function antagonistically on *COOLAIR* processing and *FLC* regulation. In *Arabidopsis*, the isolation of loss-of-function mutations of the NTC/NTR complex has revealed differential regulation by different components even on the same target *FLC*. For example, a mutation on *PRP8* causes elevated *FLC* level (30), while a mutation on *BRR2* reduces *FLC* expression (41). One could argue this is through indirect effects of the complex; however, we propose that the NTC/NTR complex functions as the nexus determining which cotranscriptional complexes associate with RNA Pol II state (with feedback between these steps). We propose that AGO1 and the THO/TREX complex are differentially recruited by the NTC/NTR to influence the cotranscriptional coupling of the spliceosome to RNA Pol II. At *FLC/COOLAIR* this links to delivery of a local chromatin environment that determines *FLC* transcriptional initiation and elongation rate (Fig. 5). In this respect, the antagonistic cotranscriptional complexes conferring quantitative transcriptional regulation are like a tug of war between RNA Pol II-associating complexes.

The mechanistic basis for this tug of war between AGO1 and THO/TREX requires additional analysis. AGO1 and the THO/TREX physically interact with NTC components in the nucleus and may associate with different Pol II states, which determines the differential processing of RNA. Nevertheless, we do not rule out the possibility that they function together at some targets as shown in *trans*-acting siRNA biogenesis (42, 43). In regard to the cotranscriptional silencing mechanism, a classic Argonaute protein’s involvement is in an RNAi-mediated heterochromatin-silencing mechanism, elegantly elaborated in fission yeast, worms, and plants (1). An important question, therefore, is whether there are sRNAs involved in *FLC* regulation. So far, only one group of Pol IV/RDR2/DCL3-dependent sRNA hybridizing to the *FLC* 3′ region has been reported, and these are highly enriched in mature siliques rather than seedlings (44). Analysis of flowering time and *FLC* expression in a range of mutants defective in sRNA production showed a *dcl1 dcl3* double mutant exhibited significantly higher *FLC* expression. Although DCL3 is not required for FCA-mediated *FLC* repression (*SI Appendix*, Fig. S5) and DCL1 is mainly required for producing miRNA (45) (*FLC* is not a miRNA target), redundant DICER activity could produce sRNA. Therefore, we searched sRNA sequence databases (http://ipf.sustech.edu.cn/pub/asrd/) (46) and found a low number of sRNA reads at the *FLC* locus, including low-abundance sRNAs complementary to the intron/exon junction region of proximal *COOLAIR* (*SI Appendix*, Fig. S9). This location was intriguing given the observed change in *COOLAIR* splicing in *ago1*. We therefore analyzed RNAs coimmunoprecipitated with AGO1 and found sRNA fragments complementary to *COOLAIR* over the proximal intron/exon junction region (*SI Appendix*, Fig. S10 *A*–*C*), where AGO1 was enriched on *COOLAIR* (Fig. 2*B*). A previous report in fission yeast had defined a class of Dicer-independent sRNA called primal sRNA (priRNA) generated from degradation products of transcripts that associate with Ago1 and target antisense transcripts (47). The priRNAs mediate an Argonaute-involved transcriptome surveillance mechanism that may be relevant to our findings. We therefore suggest a close connection between cotranscriptional RNA processing and turnover of aberrant transcripts by RNA-silencing factors. It is also possible that Argonaute proteins are recruited directly to nascent RNA or via protein–protein interactions, independently of sRNA (18, 23, 48). What recruits AGO1 to the *COOLAIR* transcript is an important question to be addressed in the future.

## Materials and Methods

### Plant Materials and Growth Conditions.

Mutant alleles *ago1-25* (29), *fca-9* (49), *fld-4* (35), *hpr1-5, tex1-4* (37), and *mac7-1* (50) were described previously. Transgenic lines *C2* (35) and *FCA-mTurquoise2* (11) were described previously. Transgenic line HPR1-GFP was described previously (37) and was provided by Chi-Kuang Wen, CAS Center for Excellence in Molecular Plant Science, Shanghai, China. AGO-HA, AGO1(DDH), AGO1(DDD), AGO1(DAH), and AGO1(ADH) were transgenic lines in *ago1-25* background, which were generously provided by James C. Carrington, Donald Danforth Plant Science Center, Olivette, MO, and were described previously (33). *dcl3* allele was provided by David C. Baulcombe, University of Cambridge, Cambridge, UK.

Seeds were surface-sterilized and sown on standard half-strength Murashige and Skoog (1/2 MS) medium plate without glucose. Plates were stratified at 4 °C for 3 d before transferred to long-day conditions (16-h light at 20 °C, 8-h darkness at 16 °).

### Expression Analysis.

Ten-day-old seedlings were harvested, and RNA was extracted. TURBO DNase (Ambion) was used to remove DNA contamination before reverse transcription. RNA was reverse-transcribed by SuperScript IV Reverse Transcriptase (Invitrogen) using gene-specific primers. qPCR analysis was performed, and data were normalized to *UBC*. Primers are described in *SI Appendix*, Table S1.

### Western Blot Analysis.

Total protein extracts from 10-d-old seedlings were prepared and immunoblot was performed as described previously (9). Anti-FCA (1:8,000 dilution, homemade) antibody and rabbit immunoglobulin G horseradish peroxidase-linked whole antibody (1:10,000 dilution, NA934; GE Healthcare) were used as primary and secondary antibody, respectively, before using chemiluminescence for detection.

### RIP.

For HPR1-GFP RIP, 3 g of 14-d-old seedlings were cross-linked in 1% formaldehyde. Nuclei were enriched using Honda buffer (20 mM Hepes, 0.44 M sucrose, 1.25% Ficoll, 2.5% Dextran T40, 10 mM MgCl_2_, 0.5% Triton X-100, 5 mM dithiothreitol [DTT], and 1× protease inhibitor mixture [Roche]) in the presence of 10 U/mL RNase inhibitor (RNaseOUT; Ambion). After resuspending the pellet in 1 mL Nuclear Lysis Buffer (50 mM Tris·HCl pH 7.5, 100 mM NaCl, 1% Triton X-100, 1 mM MgCl_2_, 0.1 mM CaCl_2_, 1× protease inhibitor mixture, and 100 U/mL RNase inhibitor), 40 U/mL DNase (Invitrogen) was added for incubation for 10 min at 37 °C. The lysate was incubated 1 h at 4 °C after adding 10 μL 10% sodium dodecyl sulfate (SDS) and 5 M NaCl. The lysate was then sonicated with Diagenode Bioruptor 10 times, 30 s on/30 s off at high setting. Soluble chromatin extracts were collected after centrifugation. GFP-trap magnetic agarose beads (ChromoTek) were prewashed with low-salt wash buffer and blocked with 0.1% bovine serum albumin and 1 mg/mL transfer RNA (tRNA) in low-salt wash buffer for 1 h before adding them into IP solution. After 2 h incubation at 4 °C, the beads were washed through high-salt washing buffer and low-salt washing buffer. The beads were suspended in RNA elution buffer (100 mM NaCl, 50 mM Tris·HCl, pH 7.0, 1 mM ethylenediaminetetraacetic acid [EDTA], and 1% SDS) at 95 °C for 15 min then were reverse cross-linked at 65 °C for 1 h in the presence of Protease K and RNase inhibitor. RNA was extracted using TRIzol and subjected to reverse transcription using gene-specific primers. qPCR was performed and data were presented as IP/1% input. UBC was used as a negative control. Primers are listed in *SI Appendix*, Table S1.

HA-AGO1 RIP was performed as described previously (9). Nuclei were enriched through cross-linked seedlings. After sonication, nuclear extract was incubated with anti-HA magnetic beads (88836; Thermo Scientific) for 2 h at 4 °C. After wash, the immunoprecipitates were eluted and reverse cross-linked. RNA was extracted using TRIzol and subsequent RT-qPCR was conducted. Primers are listed in *SI Appendix*, Table S1.

### Co-IP.

Four grams of 14-d-old seedlings were cross-linked in 1% formaldehyde. Nuclei were prepared using Honda buffer (described above) and suspended in RIPA buffer (50 mM Tris·HCl, 150 mM NaCl, 1% Nonidet P-40, 0.5% Sodium deoxycholate, 0.1% SDS, 1× protease inhibitor mixture). Nuclear extracts were sonicated and incubated with Benzonase for 15 min. Four micrograms of AGO1 antibody (AS09527; Agrisera) was added into the soluble extracts incubated for 2 h before adding 25 μL Dynabeads Protein A (Invitrogen) for another 1.5 h. Beads were washed three times before eluted in 1x LDS buffer.

### DRIP.

DRIP was performed as previously described (11). Briefly, 2 g 10-d-old seedlings were used to extract DNA. DNA was treated with Proteinase K overnight and extracted again using phenol/chloroform. The DNA pellet were dissolved in water and quantified with Qubit DNA quantification kit (Invitrogen). 1 μg of DNA were subjected to sonication, which was used for IP with 5 μg of S9.6 antibody (ENH001; Kerafast) overnight at 4 °C, before adding Protein G Agarose (Invitrogen) into IP solution for another 2 h. The immunoprecipitants were washed, eluted and precipitated, which then was subjected to qPCR analysis. The data were represented as normalized to 1% of input. Primers were listed in *SI Appendix*, Table S1.

### Histone ChIP.

Histone ChIP was performed as previously described with modifications (11). Protein A magnetic beads (10002D; Invitrogen), anti-H3 (ab176842; Abcam), and anti-H3K4me1 (ab176877; Abcam) were used. After immunoprecipitation, the eluted DNA was quantified by qPCR with primers listed in *SI Appendix*, Table S1. Data were normalized to 1% of input and presented as the ratio of H3K4me1 to H3.

### Chromatin-Bound RNA Preparation.

Nuclei from 2 g of 10-d-old seedlings were prepared as described before in RIP. The nuclei pellet was weighed and resuspended in an equal volume of resuspension buffer (50% glycerol, 0.5 mM EDTA, 1 mM DTT, 25 mM Tris·HCl, pH 7.5, and 100 mM NaCl). After washing twice with two volumes of UREA wash buffer (25 mM Tris·HCl, pH 7.5, 300 mM NaCl, 1 M urea, 0.5 mM EDTA, 1 mM DTT, and 1% Tween 20) via pipetting up and down, the chromatin pellet was spun down, which then was rinsed gently with TES. The RNA was extracted using TRIzol.

### Microscopy.

The seeds carrying heterozygous *ago1-25* and homozygous *FCA-mTurquoise2* were sown and the 7-d-old seedlings were segregated into two populations. One population which developed normally was referred to genotype carrying wild-type AGO1 and the other with developmental defects (unexpanded dark green cotyledons) was referred to genotype carrying *ago1-25* homozygous mutation. Root tips were imaged as described previously (11). The Analyze Particles tool was applied to obtain “area” data for each condensate after manual thresholding by ImageJ software. The same settings were used on all images.

### Mass Spectrometry.

Fine powder from 14-d-old seedling was dissolved in IP buffer (50 mM Tris·HCl, pH 8.0, 150 mM NaCl, 1% Triton X-100, and 1× protease inhibitor mixture). Extracts were immunoprecipitated with GFP-trap magnetic agarose beads (ChromoTek). The affinity-purified protein samples were separated on gel. The gel slices were washed with 50 mM TEAB buffer, pH 8 (Sigma) and incubated with 10 mM DTT for 30 min at 65 °C followed by incubation with 30 mM iodoacetamide at room temperature (both in 50 mM TEAB). After washing and dehydration with acetonitrile, the gels were soaked with 50 mM TEAB containing 10 ng/µL Sequencing Grade Trypsin (Promega) and incubated at 50 °C for 8 h. The eluted peptide solution was dried down, and the peptides were dissolved in 0.1%TFA/3% acetonitrile. Aliquots were analyzed by nanoLC-MS/MS on an Orbitrap Fusion Tribrid mass spectrometer coupled to an UltiMate 3000 RSLCnano LC system (Thermo Fisher Scientific). Recalibrated peaklists were generated with MaxQuant 1.6.0.16 REF in LFQ mode using the TAIR10_pep_20101214 *A. thaliana* protein sequence database (https://www.arabidopsis.org/, 35,386 entries) plus the MaxQuant contaminants database (245 entries). The quantitative LFQ results from MaxQuant with default parameters were used together with search results from an in-house Mascot Server 2.4.1 (Matrixscience, London) on the same databases. The Mascot search results were imported into Scaffold 4.11 (https://www.proteomesoftware.com/) using identification probabilities of 99% for proteins and 95% for peptides.

### sRNA Fragments Detection.

One gram of Col-0 and HA-AGO1 seedlings were ground into fine powder and subjected to immunoprecipitation using anti-HA magnetic beads (88836; Thermo). The beads were then extracted with TRIzol agent (Invitrogen) to retrieve the RNAs bound to AGO1. RNAs were reverse-transcribed using a Mir-XTM miRNA First Strand Synthesis Kit (Takara) according to the manufacturer’s instructions. The complementary DNA was amplified using sRNA sequences as forward primers (designed multiple primers complementary to *COOLAIR* sequence across the proximal *COOLAIR* transcript) and the adaptor as the reverse primer. miR159 was included as a positive control. qPCR was performed using the Mir-XTM miRNA qRT-PCR SYBR Kit (Takara) on LightCycler480 II (ROCHE) and qPCR data were normalized to tRNA. Primers are listed in *SI Appendix*, Table S1.

## Supplementary Material

Supplementary File

Supplementary File

## Data Availability

Previously published data were used for this work (12, 28). All other study data are included in the article and/or supporting information.
