# Supplementary material for: Antagonistic cotranscriptional regulation through ARGONAUTE1 and the THO/TREX complex orchestrates FLC transcriptional output
Source: Proc Natl Acad Sci U S A. 2021 Nov 17;118(47):e2113757118. doi: 10.1073/pnas.2113757118 (PMC8617408; doi:10.1073/pnas.2113757118)
Supplement: Supplementary File [file pnas.2113757118.sapp.pdf]

PNAS
www.pnas.org

Supplementary Information for
Antagonistic co-transcriptional regulation through ARGONAUTE1 and the THO/TREX complex orchestrates *FLC* transcriptional output

Congyao Xu[a], Xiaofeng Fang[a,b], Tiancong Lu[a] and Caroline Dean[a]*.

*Corresponding author: Caroline Dean.
Email: caroline.dean@jic.ac.uk

This PDF file includes:

Figures S1 to S10
Table S1
Legend for Dataset S1

Other supplementary materials for this manuscript include the following:

Dataset S1

		Total protein native IP		Nulcear protein crosslinked IP	
		SDG26	AGO1	SDG26	AGO1
Col-0	#1	0	0	0	0
	#2	0	0	0	0
SDG26-GFP	#1	9	14	108	1
	#2	23	11	115	3
GFP		0	0	0	0

Figure. S1. AGO1 co-immunoprecipitates with SDG26-GFP. Table listing the number of unique peptides of SDG26 and AGO1 identified in SDG26-GFP total protein native purification and crosslinked IP. Wildtype Col-0 and a transgenic line expressing *GFP* alone were included as negative controls.

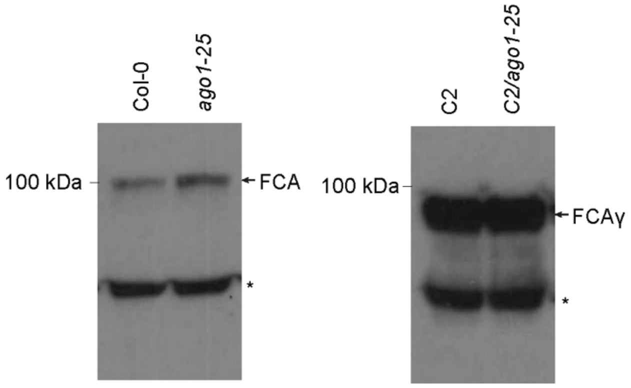

Figure. S2. Endogenous FCA or FCA γ protein level is not affected by *ago1-25*. FCA and FCA γ protein level in various genotypes determined by western blot analysis. The asterisk indicates non-specific signal.

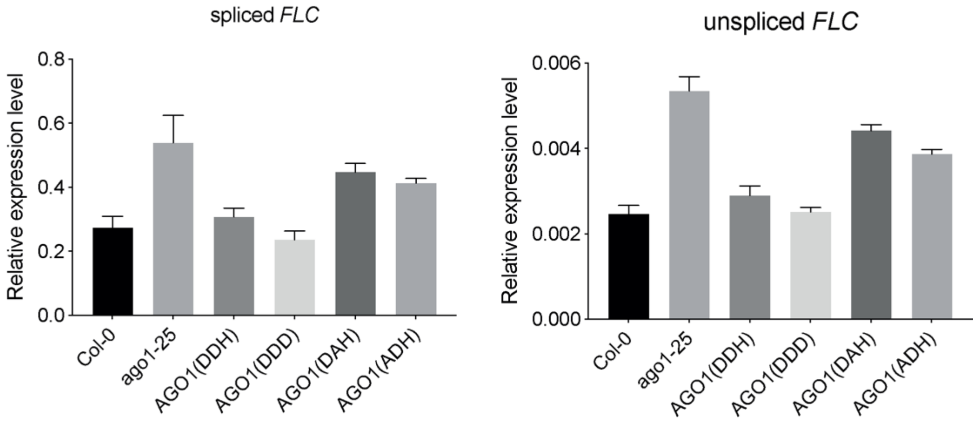

Figure. S3. Expression of spliced and unspliced *FLC* relative to *UBC* in various genotypes. AGO1(DDH) and AGO1(DDD) represent wildtype slicer. AGO1(DAH) and AGO1(ADH) represent deficient slicer. All of these lines are in *ago1-25* background. Data are presented as the mean ± s.e.m (n = 3).

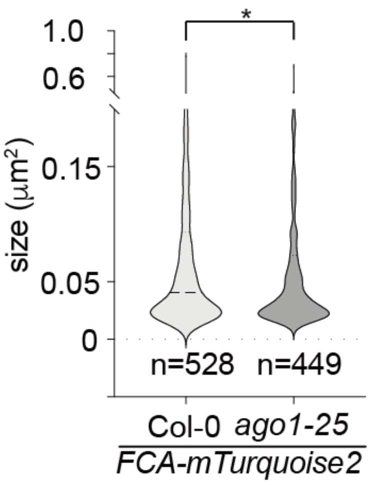

Figure. S4. The size distribution of FCA-mTurquoise2 condensates in the roots of Col-0 and *ago1-25* seedlings with the same copies of FCA-mTurquoise2 transgene. Asterisks indicate significant differences between the two groups (*P ≤ 0.0178, two-tailed *t* test).

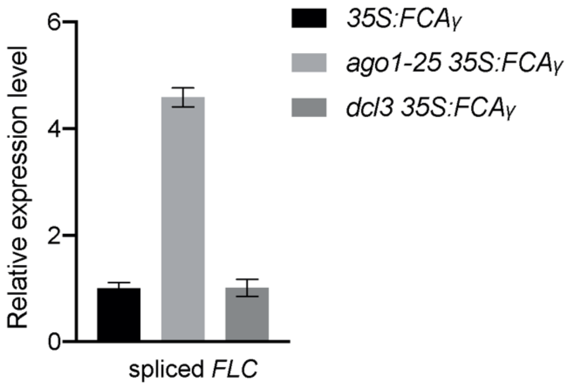

Figure. S5. DCL3 is not quired for FCA mediated *FLC* silencing. Expression of spliced *FLC* relative to *UBC* in various genotypes. Data are presented as the mean ± s.e.m (n = 3).

A

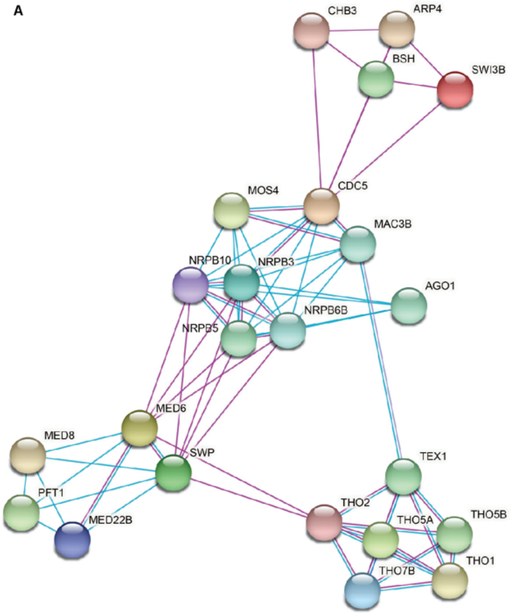

B

Identified Protein	Accession No.	Molecular Mass (kDa)	Annotation	No. of Matched Unique Peptides IP1	IP2
THO1/HPR1	AT5G09860	68 kDa	THO/TREX complex	354	458
THO2	AT1G24706	206 kDa	THO/TREX complex	353	541
THO5A	AT5G42920	93 kDa	THO/TREX complex	211	325
THO5B	AT1G45233	52 kDa	THO/TREX complex	108	168
TEX1	AT5G56130	35 kDa	THO/TREX complex	64	94
THO6	AT2G19430	40 kDa	THO/TREX complex	61	90
THO7A	AT3G02950	27 kDa	THO/TREX complex	58	79
THO7B	AT5G16790	27 kDa	THO/TREX complex	32	36
AGO1	AT1G48410	116 kDa	ARGONAUTE1	1	2
NRPB1	AT4G35800	205 kDa	RNA polymerase II subunit	0	1
NRPB2	AT4G21710	135 kDa	RNA polymerase II subunit	2	1
MOS4	AT3G18165	30 kDa	NTC complex	1	1
CDC5	AT1G09770	96 kDa	NTC complex	0	2
MAC3A	AT1G04510	57 kDa	NTC complex	0	4
MAC3B	AT2G33340	57 kDa	NTC complex	0	6
MAC7	AT2G38770	173 kDa	NTC related complex	2	6
MAC9	AT5G28740	107 kDa	NTC related complex	2	4
SYF3	AT5G41770	85 kDa	NTC related complex	0	3
MAC11	AT1G06220	110 kDa	spliceosome related	4	10
PRP17	AT1G10580	65 kDa	spliceosome related	0	1
PRP8	AT1G80070	275 kDa	spliceosome related	4	12
SAP155	AT5G64270	141 kDa	spliceosome related	0	3
U5	AT1G20960	247 kDa	spliceosome related	15	15

Figure. S6. Physical associations between AGO1, NTC/NTR, THO/TREX and RNA Pol II. (A) String analysis of nuclear AGO1 interactors. (B) Table listing the number of unique peptides of proteins identified in HPR1-GFP native immunoprecipitation. Col-0 was used as a negative control.

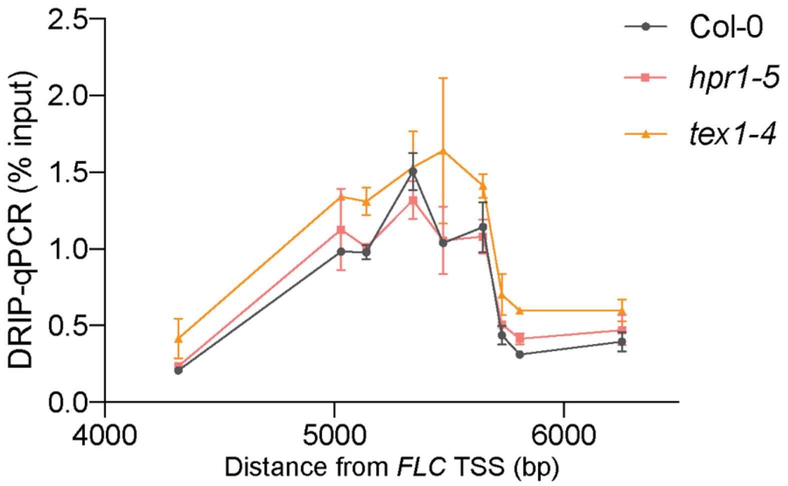

Figure. S7. *COOLAIR* R-loop is not affected in THO/TREX mutants. DRIP–qPCR determining R-loop level in various genotypes. The number on x axis is the distance to *FLC* TSS. Data are mean ± s.e.m.(n=3).

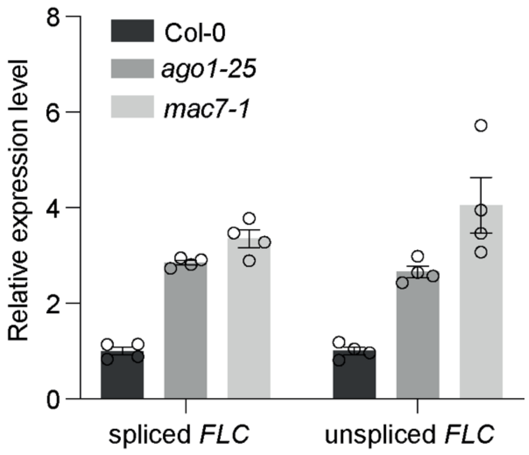

Figure. S8. MAC7 is required for *FLC* repression. Expression of spliced and unspliced *FLC* relative to *UBC* in various genotypes. Data are presented as the mean ± s.e.m (n = 4).

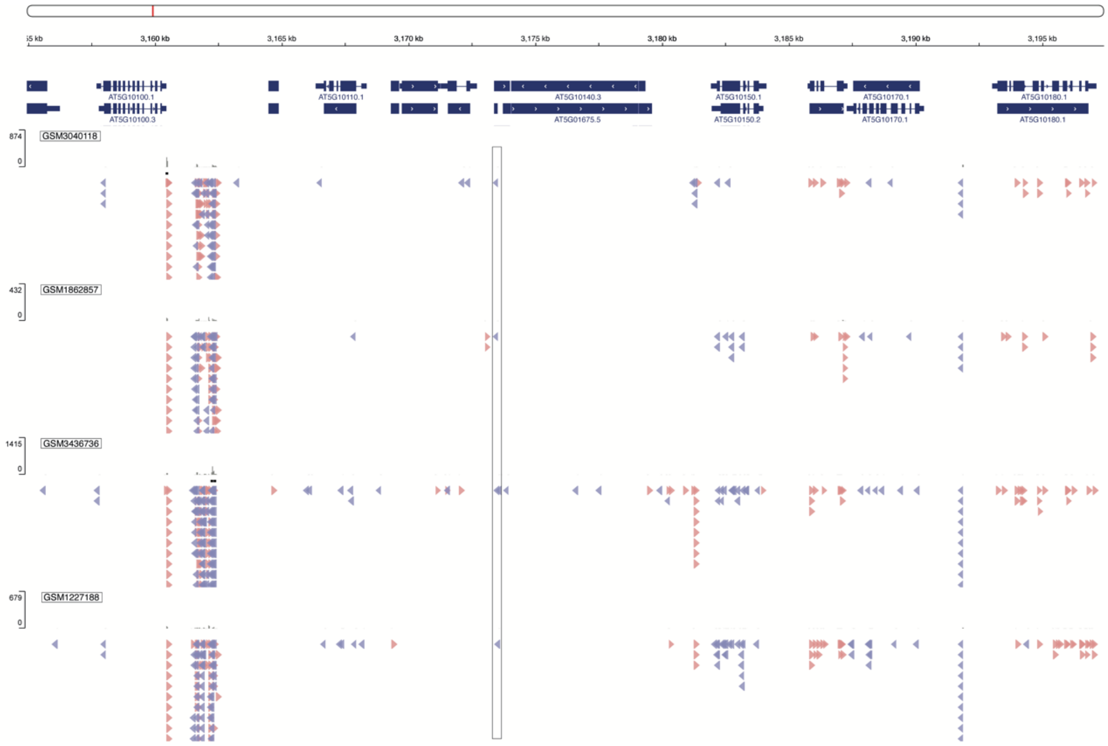

Figure. S9. A snapshot of sRNA over the *FLC* locus (AT5G10140) from sRNA sequencing databases. The rectangle in the middle marks the sRNA reads complementary to *COOLAIR* (AT5G01675) from four different published databases.

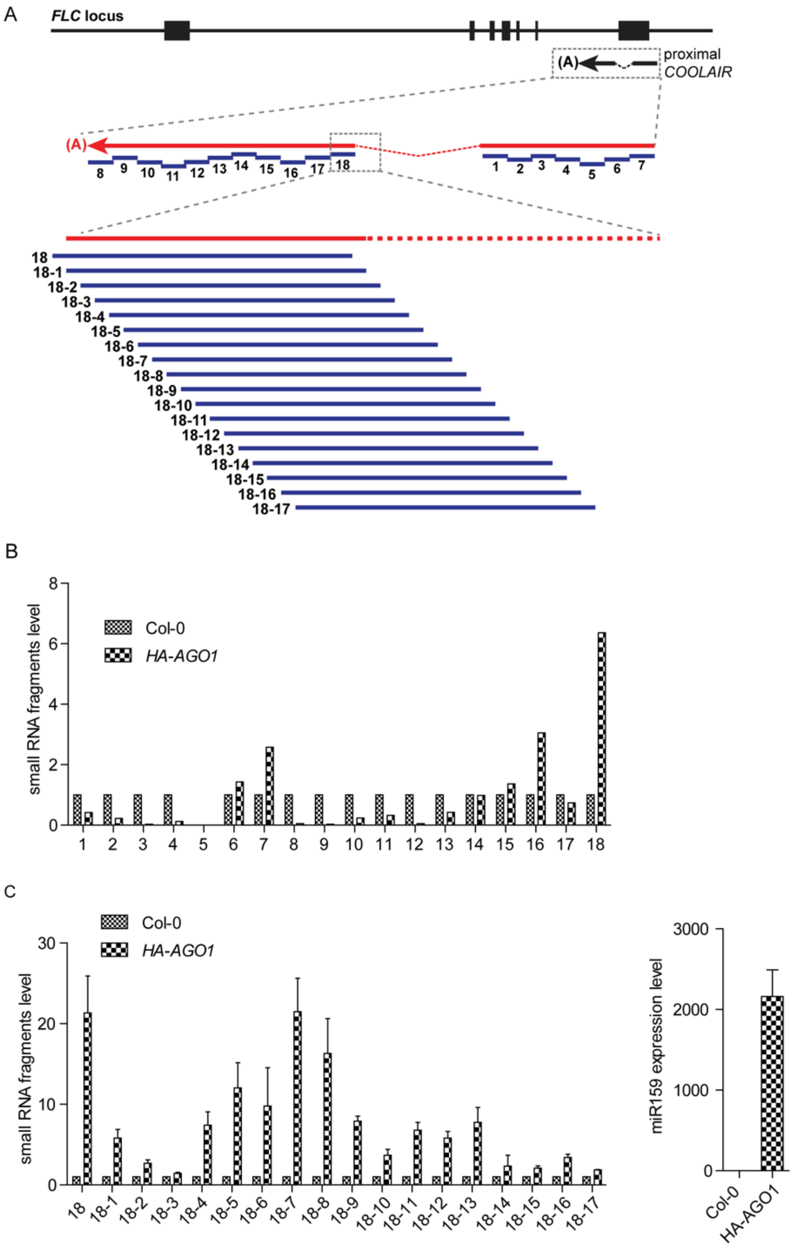

Figure. S10. Small RNA fragments complementary to *COOLAIR* were enriched over proximal *COOLAIR* intron/exon junction region. (A) A schematic of small RNA fragments mapping over the proximal *COOLAIR* transcript. Navy bars represent forward primers used for cDNA synthesis (see Materials and Methods). Red parts in the schematic represent enlargements of the grey boxes with dashed lines. (B) A rough mapping of small RNA fragments complementary to proximal *COOLAIR* based on RT-qPCR analysis. The positions of the forward primers for amplicons 1-18 refer to the schematic in (A). Data was normalized to Col-0. (C) A fine mapping of small RNA fragments complementary to proximal *COOLAIR* intron/exon junction region based on RT-qPCR analysis. The positions of the forward primers for amplicons 18 and 18-1 to 18-17 refer to the schematic in (A). miR159 expression level was included as a positive control. Data were normalized to Col-0. Data are mean ± s.e.m.(n=3).

Table S1. Primers used in this study.

Primers used for expression analysis		
**Primer position**	**Sequence 5'-3'**	**Note**
UBC_F	CTGCGACTCAGGGAATCTTCTAA	
UBC_R	TTGTGCCATTGAATTGAACCC	for RT
FLC_spliced_F	AGCCAAGAAGACCGAACTCA	
FLC_spliced_R	TTTGTCCAGCAGGTGACATC	for RT
FLC unspliced_F	CGCAATTTTCATAGCCCTTG	
FLC unspliced_R	CTTTGTAATCAAAGGTGGAGAGC	for RT
set2_F	TCATCATGTGGGAGCAGAAG	proximal *COOLAIR*
set2_R	TCTCACACGAATAAGGTGGCTA	
set1_LP	TGGTTGTTATTTGGTGGTGTG	for RT
set4_F	GTATCTCCGGCGACTTGAAC	distal *COOLAIR*
set4_R	GGATGCGTCACAGAGAACAG	
FLC-158_F	GCCCGACGAAGAAAAAGTAG	for RT
**Primers used for AGO1-HA RIP-qPCR**		
**Primer position (relative to FLC TSS)**	**Sequence 5'-3'**	**Note**
a_F	CTGTTCTCTGTGACGCATCC	for RT
a_R	AGGGGGAACAAATGAAAACC	
b_F	AGTTTGGCTTCCTCATACTTATGG	for RT
b_R	CAATGAACCTTGAGGACAAGG	
c_F	TGAAATGTTACGAATACTAGCGTGT	for RT
c_R	GGATCAAAACTACTAGCTAACCCTTG	
d_F	AGAACAACCGTGCTGCTTTT	for RT
d_R	TGTGTGCAAGCTCGTTAAGC	
e_F	CCGGTTGTTGGACATAACTAGG	for RT
e_R	CCAAACCCAGACTTAACCAGAC	
f_F	GCTTCCAAACTTAAAAGCTTAAACA	for RT
f_R	TCTTTTTGTCTTCTATCCAAGGAAT	
g_F	AGATTATAGATACTGCTTCCAAACT	for RT
g_R	TTCACACCACCAAATAACAAC	
h_F	CACCTTAAATCGGCGGTTG	for RT
h_R	TACAAACGCTCGCCCTTATC	
i_F	GATATGTAATTATTCCGCTGATAAGG	for RT
i_R	TCTTGGCCAAAGAGAGAGTATT	
j_F	CGTGTGAGAATTGCATCGAG	for RT
j_R	AAAAACGCGCAGAGAGAGAG	
**Primers used for ChIP (H3K4me1)**		
**Primer position (relative to FLC TSS)**	**Sequence 5'-3'**	**Note**
FLC_-392_F	ACTATGTAGGCACGACTTTGGTAAC	
FLC_-249_R	TGCAGAAAGAACCTCCACTCTAC	
FLC_-49_F	GCCCGACGAAGAAAAAGTAG	
FLC_58_R	TTCAAGTCGCCGGAGATACT	
FLC_581_F	TTTTGTTCATTTCTCTCTCT	
FLC_672_R	AAACTTCACTCAACAACATC	
FLC_1533_F	TTGACAATCCACAACCTCAATC	
FLC_1670_R	TCAATTTCCTAGAGGCACCAA	
FLC_2465_F	AGTTTGGCTTCCTCATACTTATGG	
FLC_2579_R	CAATGAACCTTGAGGACAAGG	
FLC_3197_F	GGGGCTGCGTTTACATTTTA	
FLC_3353_R	GTGATAGCGCTGGCTTTGAT	
FLC_3643_F	TGAAATGTTACGAATACTAGCGTGT	
FLC_3752_R	GGATCAAAACTACTAGCTAACCCTTG	
FLC_5030_F	CCGGTTGTTGGACATAACTAGG	
FLC_5153_R	CCAAACCCAGACTTAACCAGAC	
FLC_5672_F	CCTGCTGGACAAATCTCCGA	
FLC_5757_R	GGATTTTGATTTCAACCGCCGA	
FLC_5970_F	CGTGTGAGAATTGCATCGAG	
FLC_6088_R	AAAAACGCGCAGAGAGAGAG	
**Primers used for HPR1-GFP RIP-qPCR (*FLC*)**		
**Primer position (relative to FLC TSS)**	**Sequence 5'-3'**	**Note**
I_F	ATTAGGGCACAAAGCCCTCT	
I_R	CGACGTTTGGAGAAGGTGAC	for RT
II_F	AGCCAAGAAGACCGAACTCA	
II_R	TTTGTCCAGCAGGTGACATC	for RT
III_F	CCTGCTGGACAAATCTCCGA	
III_R	GGATTTTGATTTCAACCGCCGA	for RT
IV_F	GATATGTAATTATTCCGCTGATAAGG	
IV_R	TCTTGGCCAAAGAGAGAGTATT	for RT
**Primers used for HPR1-GFP RIP-qPCR (*COOLAIR*)**		
**Primer position (relative to FLC TSS)**	**Sequence 5'-3'**	**Note**
3643_F	TGAAATGTTACGAATACTAGCGTGT	for RT
3752_R	GGATCAAAACTACTAGCTAACCCTTG	
5030_F	CCGGTTGTTGGACATAACTAGG	for RT
5153_R	CCAAACCCAGACTTAACCAGAC	
5442_F	AGATTATAGATACTGCTTCCAAACT	for RT
5553_R	TTCACACCACCAAATAACAAC	
5672_F	CCTGCTGGACAAATCTCCGA	for RT
5757_R	GGATTTTGATTTCAACCGCCGA	
5792_F	GATATGTAATTATTCCGCTGATAAGG	for RT
5858_R	TCTTGGCCAAAGAGAGAGTATT	
5948_F	CGTGTGAGAATTGCATCGAG	for RT
6088_R	AAAAACGCGCAGAGAGAGAG	
6066_F	CTCTCTCTCTGCGCGTTTTT	for RT
6171_R	ATTGGGCCGAATGTGACCGA	
**Primers used for DRIP-qPCR**		
**Primer position**	**Sequence 5'-3'**	**Note**
FLC_4322_F	AGAACAACCGTGCTGCTTTT	
FLC_4469_R	TGTGTGCAAGCTCGTTAAGC	
FLC_5342_F	TTTTTGTTATGGTTAGGTTTGGA	
FLC_5411_R	AGTAGCACTACTTCTAGACACTTGGA	
FLC_5472_F	GCTTCCAAACTTAAAAGCTTAAACA	
FLC_5600_R	TCTTTTTGTCTTCTATCCAAGGAAT	
FLC_5648_F	TAATCATCATGTGGGAGCAG	
FLC_5727_R	GGAGAGTCACCGGAAGATTG	
FLC_5730_F	CACCTTAAATCGGCGGTTG	
FLC_5814_R	TACAAACGCTCGCCCTTATC	
FLC_5948_F	CGTGTGAGAATTGCATCGAG	
FLC_6088_R	AAAAACGCGCAGAGAGAGAG	
FLC_6768_F	TTGTAAAGTCCGATGGAGACG	
FLC_6838_R	ACTCGGCGAGAAAGTTTGTG	
**Primers used for short RNA fragments detection**		
**Primer**	**Sequence 5'-3'**	**Note**
sR1 F	TTATTCGTGTGAGAATTGCAT	
sR2 F	TCGAGATCTTGAGTGTATGTG	
sR3 F	TGTTCTTCACTTCTGTCAAAA	
sR4 F	TTGTGTTTGCTTCACAGTGAA	
sR5 F	TGAAGAAGCCTACGGCTTATTT	
sR6 F	TTGCAACAGGGACGTGGCTCT	
sR7 F	TCTCTCTCTCTCTCTGCGCGT	
sR8 F	TACTGCTTCCAAACTTAAAAG	
sR9 F	TTAAACATAAAGAACACACAA	
sR10 F	TTATGAGAAACATAACCTTCT	
sR11 F	TAATGGTTGTTATTTGGTGGT	
sR12 F	TGAAAAAGATATTCCTTGGAT	
sR13 F	TAGTGATTTTGACCTATGATT	
sR14 F	TCGTACAGATGGAGAATAATC	
sR15 F	TCATGTGGGAGCAGAAGCTGA	
sR16 F	TGGAGATGTCACCTGCTGGAC	
sR17 F	TCTCCGACAATCTTCCGGTGA	
sR18 F	TCTCCCACTACTTAATTAGCC	
sR18-1 F	TCCCACTACTTAATTAGCCAC	
sR18-2 F	CCCACTACTTAATTAGCCACC	
sR18-3 F	CCACTACTTAATTAGCCACCT	
sR18-4 F	CACTACTTAATTAGCCACCTT	
sR18-5 F	ACTACTTAATTAGCCACCTTA	
sR18-6 F	CTACTTAATTAGCCACCTTAA	
sR18-7 F	TACTTAATTAGCCACCTTAAA	
sR18-8 F	ACTTAATTAGCCACCTTAAAT	
sR18-9 F	CTTAATTAGCCACCTTAAATC	
sR18-10 F	TTAATTAGCCACCTTAAATCG	
sR18-11 F	TAATTAGCCACCTTAAATCGG	
sR18-12 F	AATTAGCCACCTTAAATCGGC	
sR18-13 F	ATTAGCCACCTTAAATCGGCG	
sR18-14 F	TTAGCCACCTTAAATCGGCGG	
sR18-15 F	TAGCCACCTTAAATCGGCGGT	
sR18-16 F	AGCCACCTTAAATCGGCGGTT	
sR18-17 F	GCCACCTTAAATCGGCGGTTG	
tRNA F	TAGGACACTAGACTCTGAATC	

Dataset S1 (separate file). List of proteins identified by HPR1-GFP affinity purifications.
